# Mobilizing Vacuolar Sugar Increases Vegetative Triacylglycerol Accumulation

**DOI:** 10.3389/fpls.2021.708902

**Published:** 2021-08-12

**Authors:** Sanket Anaokar, Hui Liu, Jantana Keereetaweep, Zhiyang Zhai, John Shanklin

**Affiliations:** Brookhaven National Laboratory, Biology Department, Upton, NY, United States

**Keywords:** triacylglycerol, fatty acid synthesis, sugar flux, sugar compartmentalization, sugar transport

## Abstract

Photosynthetically derived sugars provide carbon skeletons for metabolism and carbon signals that favor anabolism. The amount of sugar available for fatty acid (FA) and triacylglycerol (TAG) synthesis depends on sugar compartmentation, transport, and demands from competing pathways. We are exploring the influence of sugar partitioning between the vacuole and cytoplasm on FA synthesis in Arabidopsis by building on our previous finding that reduced leaf sugar export in the sucrose-proton symporter2 (*suc2*) mutant, in combination with impaired starch synthesis in the ADP-glucose pyrophosphorylase (*adg1*) mutant, accumulates higher sugar levels and increased total FA and TAG compared to the wild type parent. Here we sought to relocalize sugar from the vacuole to the cytoplasm to drive additional FA/TAG synthesis and growth. Arabidopsis *suc2 adg1* was therefore crossed with tonoplast monosaccharide transporter mutants *tmt1* and *tmt2* and overexpression of the sucrose/proton cotransporter *SUC4* in which *tmt1 tmt2* impairs sugar transport to the vacuole from the cytoplasm and *SUC4* overexpression enhances sugar transport in the reverse direction from the vacuole to the cytoplasm. A resulting homozygous *suc2 adg1 tmt1 tmt2 SUC4* line was used to test the hypothesis that increased intracellular carbon supply in the form of sugars would increase both FA and TAG accumulation. The data shows that relative to *suc2 adg1, suc2 adg1 tmt1 tmt2 SUC4* significantly increases leaf total FA content by 1.29-fold to 10.9% of dry weight and TAG by 2.4-fold to 2.88%, supporting the hypothesis that mobilizing vacuolar sugar is a valid strategy for increasing vegetative oil accumulation.

## Introduction

Maintaining sugar homeostasis in the cell requires coordinated control between production, distribution, storage, and metabolism of sugar, which is essential for plant growth and its development. In plants, photosynthetically derived sugars get exported from the production site (source tissues) to consumption sites (sink tissues) where it is either used for growth and development or converted to storage compounds (Julius et al., 2017). In higher plants, sucrose is the predominant sugar for long distance transport through the phloem (Julius et al., 2017). Several decades of studies on sugar transport have identified more than 60 putative monosaccharide transporters in plants (Wormit et al., 2006). Vacuoles occupy as much as 90% of the cell by volume and are capable of both short- and long-term sugar storage. Tonoplast membranes that surround the vacuole act as a semi-permeable barrier that regulate the flux of organic and inorganic molecules between the cytoplasm and the vacuole. Several sugar transporters and proton exchange pumps are located at the tonoplast, including: tonoplast monosaccharide transporters (TMT also known as TST), plant sucrose/ proton cotransporters (SUT, defined as SUC4 in this study), vacuolar glucose transporters (VGT), early response to dehydration like (ERDL) transporters and Sugars Will Eventually be Exported Transporters (SWEET) (Wormit et al., 2006; Riccioni et al., 2008; Schulz et al., 2011; Hedrich et al., 2015; Julius et al., 2017; Jeena et al., 2019). In leaves, vacuolar sugar levels increase during the light, and decrease during the dark period (Wormit et al., 2006). Once sugar is exported from the vacuole it can be utilized either for cellular processes including growth and development or for the synthesis of starch or lipids.

In seeds, photosynthetically derived sugars provide the carbon skeletons for fatty acid (FA) and triacylglycerol (TAG) synthesis (Xu and Shanklin, 2016). All plant cells are capable of synthesizing FA, which serve as important building blocks for both membranes and TAG, but vegetative (source) tissues generally accumulate no more than 0.1% of TAG per dry weight (DW), and instead store sugars and starch, which are lower in energy than lipids. Triacylglycerols (TAG) are important energy dense molecules that contribute to human nutrition, fodder for livestock, and industrial uses including biodiesel production (Xu and Shanklin, 2016). The regulation of FA synthesis and TAG assembly involves multiple steps that have been described as Push, Pull and Protect factors (Vanhercke et al., 2014). While plant vegetative tissues produce sugars and store carbon primarily as carbohydrates, they have the capacity to synthesize FAs for membrane synthesis and therefore have the metabolic potential to produce and accumulate TAGs.

A current focus of our research is to divert carbon flux from carbohydrates toward lipid accumulation. For example, a multi-gene engineering approach was used to engineer transgenic sugarcane overexpressing genes for TAG accumulation whilst simultaneously downregulating starch biosynthesis. To achieve this, WRINKLED1 (*WRI1)*, diacylglycerol acyltransferase1-2 (*DGAT1-2)* and oleosin1 (*OLE1)* were constitutively co-expressed, while subunits of the peroxisomal ABC transporter1 (*PXA*1), and the first committed step in starch synthesis mediated by ADP-glucose pyrophosphorylase (*AGP*ase) were simultaneously co-repressed in sugarcane. TAG levels in leaves of the transgenic line increased by 95-fold compared to WT (Zale et al., 2016).

Sucrose-proton symporter2, encoded by *SUC2*, is responsible for loading of sucrose into the phloem for the long-distance transport of sugar from leaves to sink tissues (Srivastava et al., 2008). Mutation of *SUC2* in *Arabidopsis* reduces sugar availability in sink tissues which leads to stunted growth (Srivastava et al., 2008). As described above, *ADG1* encodes the small subunit of ADP-glucose pyrophosphorylase (Wang et al., 1998), mutations of which prevent the storage of sugar as starch in leaf tissues (Zhai et al., 2017b). Combining *suc2* and *adg1* mutations in Arabidopsis produced a double mutant that is a starchless but which partially rescues the stunted growth of the *suc2* mutant (Zhai et al., 2017b). Also, *suc2 adg1* accumulates higher amounts of soluble sugars in its leaves compared with WT. The high sugar content in leaves of *suc2 adg1* resulted in increases of total FA and TAG content from 4.2% DW and 0.1% DW to 8% DW and 1.2% of DW, respectively (Zhai et al., 2017b).

Building on our successful strategy of *suc2 adg1* in promoting vegetative TAG accumulation, in the current study, we sought to relocalize sugar from the vacuole to the cytoplasm to drive further increases in FA and TAG synthesis by manipulating sugar transport across the tonoplast membrane by modulating the expression of genes encoding TMTs and SUC4. We first constructed *tmt1 tmt2 SUC4* by crossing *tmt1 tmt2* and *SUC4* and the resulting *tmt1 tmt2 SUC4* line was crossed with *suc2 adg1* to generate the quadruple mutant *suc2 adg1 tmt1 tmt2* that also overexpresses *SUC4*. As described above, mutating *TMT1* and *TMT2* reduces the transport of sugar from the cytoplasm to the vacuole for storage, and overexpressing *SUC4* mobilizes sugar reserves from the vacuole to cytoplasm. While other transporters and passive processes may contribute to vacuolar compartmentalization of sugar, our strategy was to modulate expression of these well-defined factors to increase cytoplasmic sugar content at the expense of vacuolar sugar while minimizing export via the phloem and interrupting the flow of carbon to starch. Stacking *tmt1 tmt2 SUC4* in the *suc2 adg1* background resulted in plants with larger roots and shoots as compared to *suc2 adg1*. The starchless *suc2 adg1 tmt1 tmt2 SUC4* showed a significant decrease in soluble sugar content compared to *suc2 adg1* along with increases in FA and TAG content from 4.6% DW/ 0.2% DW for WT and 8.4% DW/ 1.2% DW for *suc2 adg1* to 10.9% DW/2.88% DW, respectively. Also, the *suc2 adg1 tmt1 tmt2* SUC4 showed a significant increase in FA synthesis as compared to the WT and intermediate genotypes.

## Results

### Combining *tmt1 tmt2 SUC4* With *suc2 adg1* Partially Rescues Its Dwarf Phenotype

**Figure 1:**
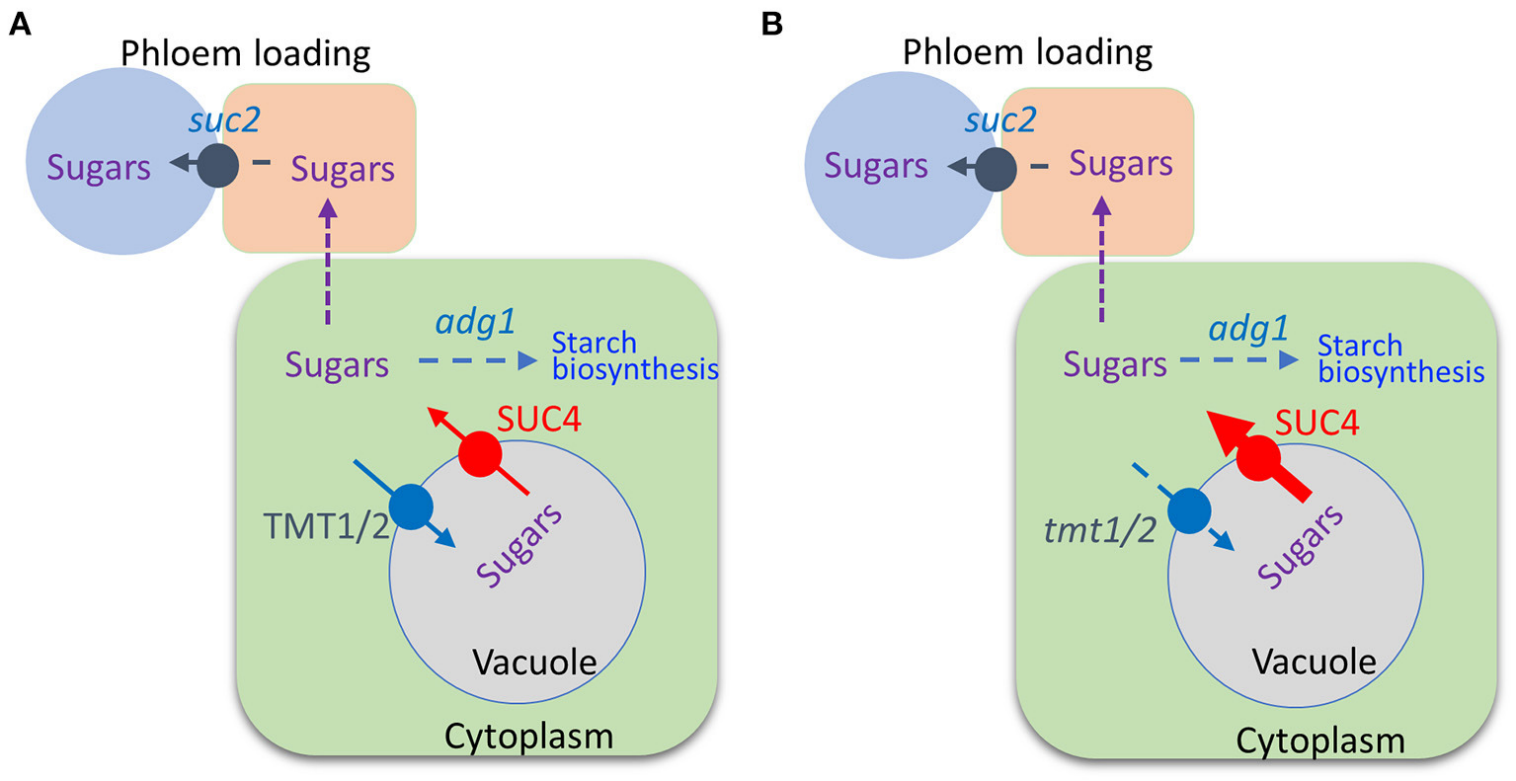
Schematic of the suc*2 adg1* and *suc2 adg1 tmt1 tmt2 SUC4* genotypes. In *suc2 adg1*, the pathway for phloem loading, and starch biosynthesis are impaired, **(A)**. In *suc2 adg1 tmt1 tmt2 SUC4*, in addition to attenuation of phloem loading and starch biosynthesis, mutations of two tonoplast monosaccharide transporters along with overexpression of the sucrose/proton cotransporter relocalize sugars from the vacuole to the cytoplasm, **(B)**.

**Figure 2:**
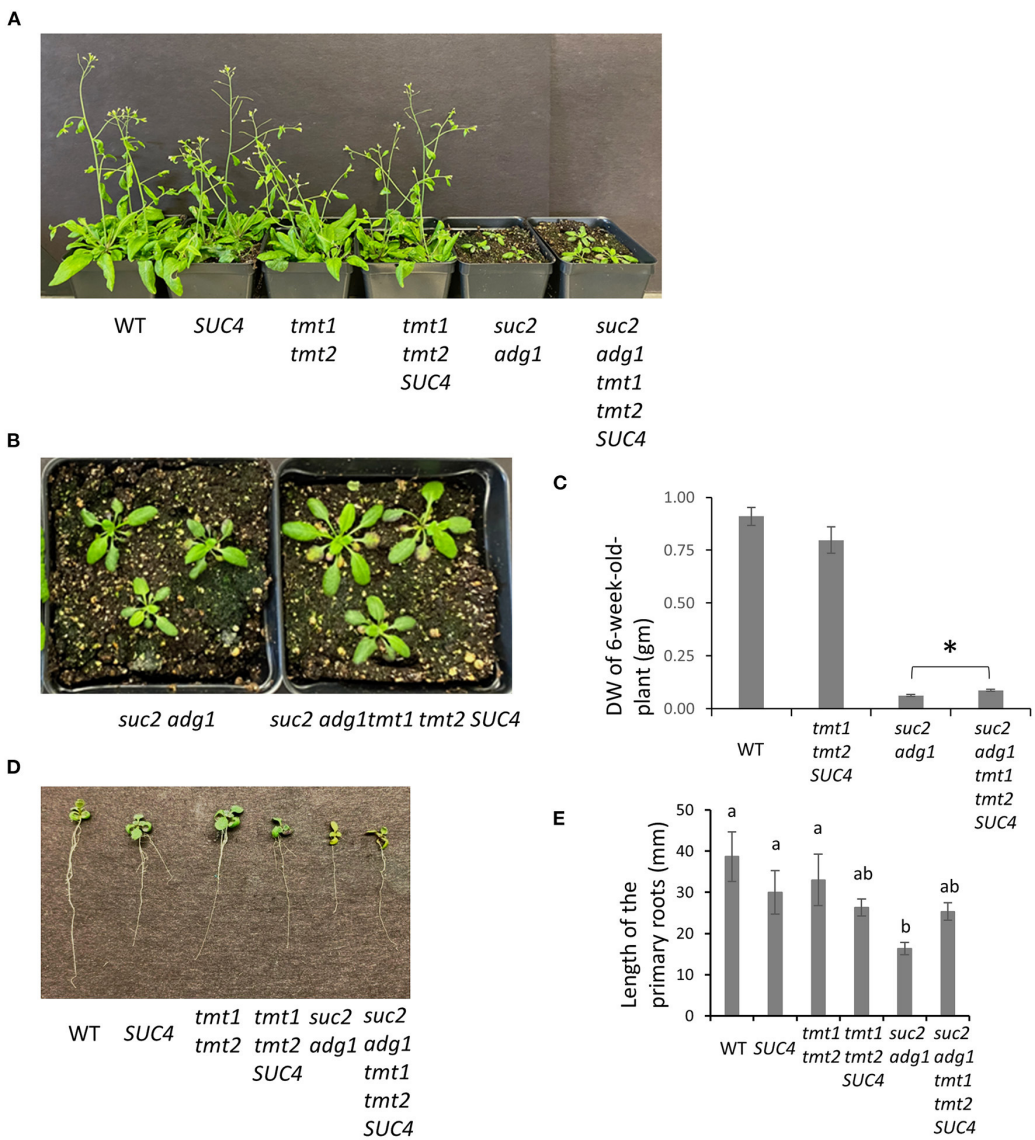
*suc2 adg1 tmt1 tmt2 SUC4* shows partial rescue of the *suc2 adg1* dwarf phenotype. **(A)** Comparison of growth of 5-week-old soil-grown WT, *SUC4, tmt1 tmt2, tmt1 tmt2 SUC4, suc2 adg1*, and *suc2 adg1 tmt1 tmt2 SUC4* plants. **(B)** Rosette leaves of *suc2 adg1* and *suc2 adg1 tmt1 tmt2 SUC4* in **(A)**. **(C)** Quantification of the DW for 6-week-old-soil grown WT*, tmt1 tmt2 SUC4, suc2 adg1*, and *suc2 adg1 tmt1 tmt2 SUC4* plants. Data represents average of 24 plants ± SD. Student's *t*-test was performed to determine the significance indicated by * (*n* = 24, *P* = 0.0015). **(D)** Representative root growth of 21-day old plate-grown WT, *SUC4, tmt1 tmt2, tmt1 tmt2 SUC4, suc2 adg1*, and *suc2 adg1 tmt1 tmt2 SUC4* plants. **(E)** Quantification of primary root length in **(D)**. Data represents average of 30 plants ± SD. Different letters indicate statistical significance between means of different genotypes established using one-way ANOVA with Tukey's mean test (*n* = 30, *P* < 0.007). Each experiment was repeated three times and data for a single representative experiment are presented.

We previously reported that combining the *adg1* and *suc2* mutations partially offset the *suc2* stunted phenotype enabling the double mutant to grow longer primary roots compared to those of *suc2* (Zhai et al., 2017b). We further crossed *suc2 adg1* double mutant with a *tmt1 tmt2 SUC4* that was obtained by crossing *tmt1 tmt2* (Wingenter et al., 2010) with *SUC4* (Schulz et al., 2011) to create a quad-mutant that also overexpressed *SUC4* (*suc2 adg1 tmt1 tmt2 SUC4*). See Figure 1 for a schematic of sugar relations in *suc2 adg1* and *suc2 adg1 tmt1 tmt2 SUC4* genotypes. The *tmt1 tmt2 SUC4* line grows similarly to WT. The *suc2 adg1 tmt1 tmt2 SUC4* showed further additional incremental reversion of the *suc2 adg1* stunted growth phenotype (Figures 2A–C) with a significant increase of 50%. This phenotype reversal was distinguishable both at the seedling stage and in mature plants. Specifically, the length of 10-day old primary roots for suc*2 adg1 tmt1 tmt2 SUC4* increased to 25 mm from 16 mm for the *suc2 adg1* seedlings (Figures 2D,E).

### Stacking of *tmt1 tmt2 SUC4* With *suc2 adg1* Decreases Its Soluble Sugar Content While Starch Content Remained Unchanged

**Figure 3:**
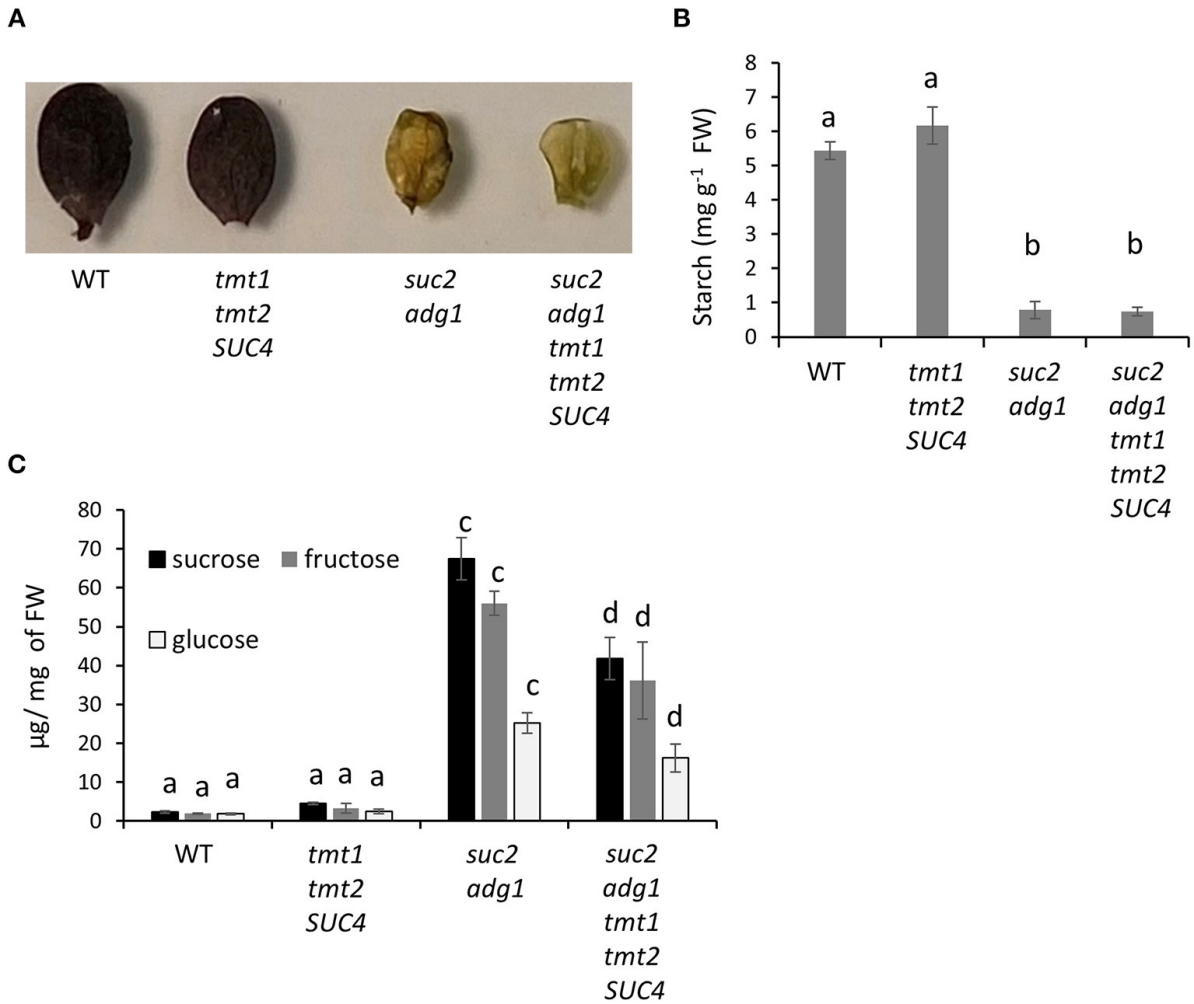
Starchless *suc2 adg1 tmt1 tmt2 SUC4* mutant shows decreased soluble sugar content compared to *suc2 adg1*. **(A)** Leaf samples from the equivalent developmental stage of WT*, tmt1 tmt2 SUC4, suc2 adg1*, and *suc2 adg1 tmt1 tmt2 SUC4* were stained with iodine to visualize their starch content. **(B)** Starch was quantitated in the same samples using corn starch as standard. **(C)** Glc, Suc, and Fru content was measured in 4-week-old soil-grown plants. For quantitation in **(B,C)**, Values represent averages of three biological replicates ± SD. Different letters represent statistical significance of differences between means using one-way ANOVA with Tukey's mean test (*N* = 3, *P* < 0.05). Each experiment was repeated three times and data for a single representative experiment are presented.

Like *suc2 adg1*, the *suc2 adg1 tmt1 tmt2 SUC4* line did not accumulate starch in its leaves (Figure 3A). The slightly higher starch accumulation in *tmt1 tmt2 SUC4* of 6.1 mg per gram of fresh weight compared to 5.4 mg per gram of fresh weight of WT was not statistically significant. However, *suc2 adg1* and *suc2 adg1 tmt1 tmt2 SUC4* have a very significant 6.9- and 7.3-fold reduction in starch content compared to WT (Figure 3B). Leaves of the *suc2 adg1* mutant showed a combined 25-fold increase in the soluble sugar content (Glc, Suc, and Fru) relative to the WT (Zhai et al., 2017b). Addition of *tmt1 tmt2 SUC4* in the double mutant background significantly reduced its combined soluble sugar content (Glc, Suc, and Fru) from 25- to 15.6-fold that of WT. This significant decrease was observed for all three soluble sugars (Glc, Suc and Fru), singly and for the total sugar content (Figure 3C). *tmt1 tmt2 SUC4* did not show any major changes in the soluble sugar content when compared to WT (Figure 3C).

### *suc2 adg1 tmt1 tmt2 SUC4* Has Higher Total FA and TAG Accumulation Than *suc2 adg1*

**Figure 4:**
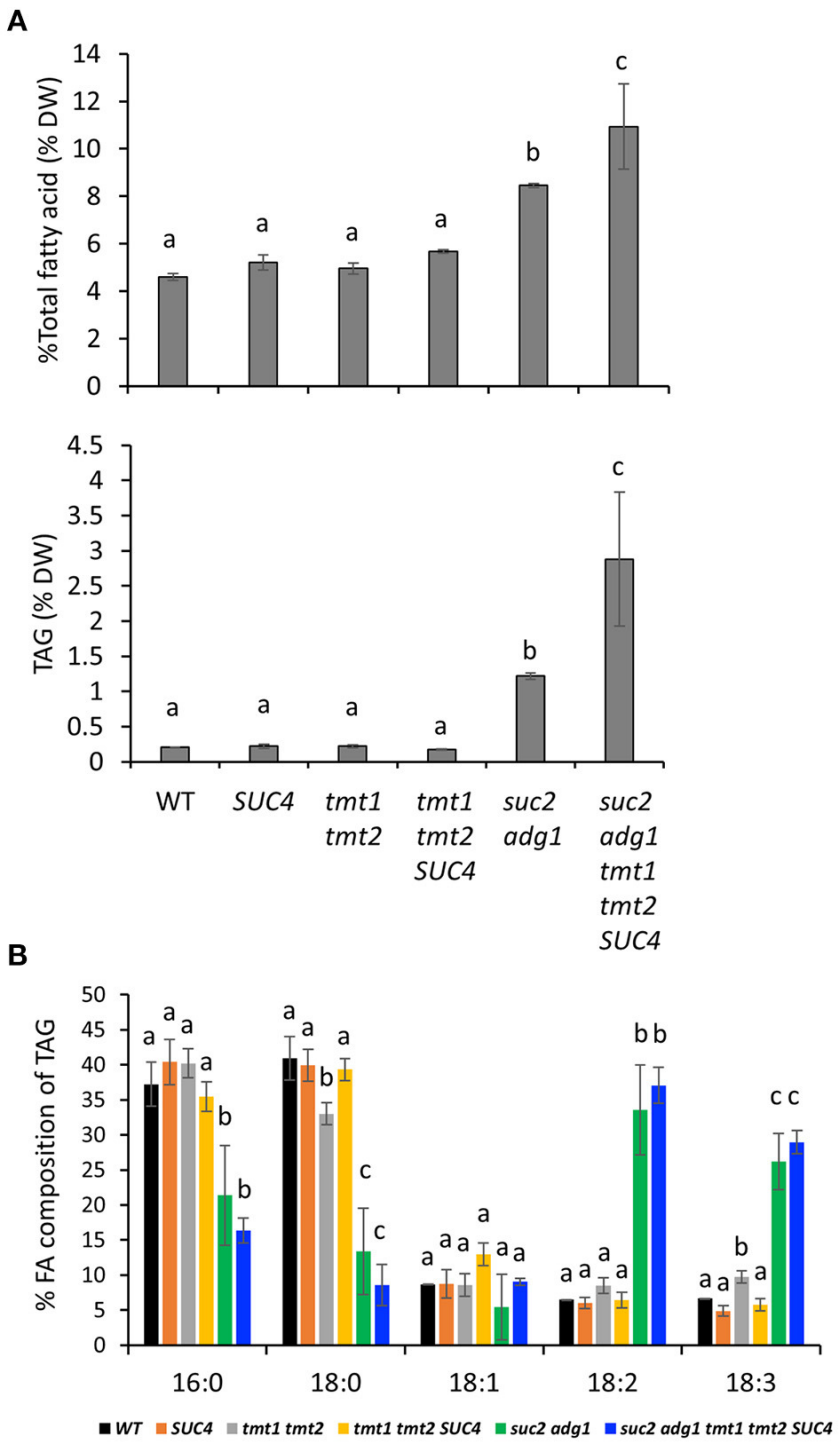
*suc2 adg1 tmt1 tmt2 SUC4* accumulates higher levels of TFA and TAG content in leaves compared to WT and the other genotypes. **(A)** Total fatty acid and TAG content of 5-week-old leaves of WT, *SUC4, tmt1 tmt2, tmt1 tmt2 SUC4, suc2 adg1*, and *suc2 adg1 tmt1 tmt2 SUC4* plants. **(B)** Fatty acid composition of 5-week-old leaves of WT, *SUC4, tmt1 tmt2, tmt1 tmt2 SUC4, suc2 adg1*, and *suc2 adg1 tmt1 tmt2 SUC4* plants. In this figure, values represent averages of three biological replicates ± SD. Different letters represent statistical significance of differences between means using one-way ANOVA with Tukey's mean test (*N* = 3, *P* < 0.05). Each experiment was repeated twice and data for a single representative experiment are presented.

*Suc2 adg1* accumulates both FA and TAG in mature leaves of 5-week old plants (Zhai et al., 2017b). As noted above, combining *tmt1 tmt2 SUC4* with *suc2 adg1* resulted in significant decrease in soluble sugar content of leaves (Figure 3C), thus we suspected this loss could be compensated by increased lipid synthesis. To test this, we compared FA and TAG accumulation across all the genotypes. The *SUC4, tmt1 tmt2* and *tmt1 tmt2 SUC4* lines showed no significant changes in FA or TAG (Figure 4A) accumulation relative to WT. Consistent with our previous findings, *suc2 adg1* showed a significant large increase in both FA content from 4.6% DW to 8.4% DW (Figure 4A) and TAG content from 0.2% DW to 1.2% DW (Figure 4A) compared to WT. However, s*uc2 adg1 tmt1 tmt2 SUC4* further significantly enhanced the FA and TAG content to 10.9% DW and 2.88% DW, respectively. The TAG FA profiles for both *suc2 adg1* and s*uc2 adg1 tmt1 tmt2 SUC4* showed large significant increases in 18:2 and 18:3 at the expense of 16:0, 18:0 and to a lesser extent, 18:1 (Figure 4B).

### *suc2 adg1 tmt1 tmt2 SUC4* Has an Elevated Rate of Lipid Synthesis Compared to WT

**Figure 5:**
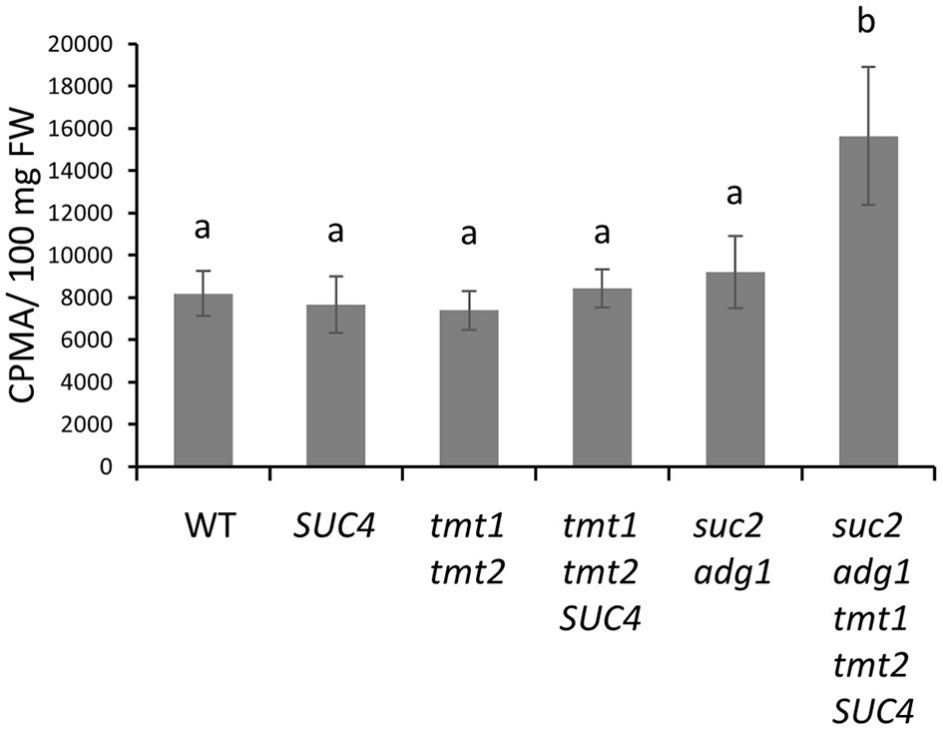
The *suc2 adg1 tmt1 tmt2 SUC4* line has the highest rate of ^14^C-acetate incorporation into lipids among the tested genotypes. Leaves of 4-week-old soil grown WT, *SUC4, tmt1 tmt2, tmt1 tmt2 SUC4, suc2 adg1*, and *suc2 adg1 tmt1 tmt2 SUC4* plants were subjected to a 30 min ^14^C acetate labeling assay. The amount of ^14^C incorporated into the total lipid fraction was estimated by liquid scintillation counting. Values represent averages of three biological replicates ± SD. Different letters represent statistical significance of differences between means using one-way ANOVA with Tukey's mean test (N=3, *P* < 0.05). Each experiment was repeated twice and data for a single representative experiment are presented.

Because stacking *tmt1 tmt2 SUC4* with *suc2 adg1* increased both its FA and TAG accumulation in leaves, we performed ^14^C-acetate labeling to investigate whether s*uc2 adg1 tmt1 tmt2 SUC4* shows a higher rate of lipid synthesis compared to WT and the other mutant lines. *SUC4, tmt1 tmt2* and *tmt1 tmt2 SUC4* mutants did not show significant changes in the rate of incorporation of label as compared to WT, though *tmt1 tmt2 SUC4* showed a 3% increase that was not significant. *suc2 adg1* followed this trend with a 12% non-significant increase in the rate of label incorporation into total lipids. In contrast, the *suc2 adg1 tmt1 tmt2 SUC4* line showed significant 91% increase in incorporation of label in total lipids relative to WT (Figure 5).

## Discussion

In previous work we reported the characterization of the *suc2 adg1* double mutant, impaired in both sugar loading into the phloem and in starch synthesis that dramatically increased leaf sugar levels by reducing its export and blocking its conversion to starch (Zhai et al., 2017b). While leaf sugar levels were increased substantially in *suc2 adg1*, we reasoned that much of the sugar would be localized in vacuoles which can occupy as much as 90% of the cell volume. To test the hypothesis that increasing cytoplasmic sugar levels would boost FA synthesis and TAG accumulation, we combined *suc2 adg1* with mutations in *tmt1, tmt2* while simultaneously overexpressing *SUC4* to relocalize sugar from the vacuole to the cytoplasm. TMT1 and TMT2 are principally responsible for the accumulation of sugar into the vacuole, and the overexpression of SUC4, which mediates sugar efflux from vacuole to cytoplasm likely contributes little to the observed sugar/TAG phenotypes in this study. Its overexpression was intended to reduce vacuolar sugar levels resulting from passive influx of sugars in the *tmt1 tmt2*-containing lines. The hypothesis is supported by the following: (1) while FA synthesis rates in *tmt1 tmt2 SUC4* and *suc2 adg1* are 3 and 12% higher than those of WT, respectively, the rate in *suc2 adg1 tmt1 tmt2 SUC4* is significantly higher at 91% than that of both *suc2 adg1* and WT. (2) lipid synthesis is boosted in *suc2 adg1 tmt1 tmt2 SUC4* as evidenced by an approximate doubling of its FA content from 4.6% DW to 10.9% DW, (3) the accumulation of TAG in *suc2 adg1 tmt1 tmt2 SUC4* is 2.3-fold that of *suc2 adg1 and* 14-fold than that WT.

It is notable that sugar contents (the sum of sucrose, fructose and glucose) for *suc2 adg1* and *suc2 adg1 tmt1 tmt2 SUC4* are 24- and 16-fold higher than WT, respectively, whereas in *tmt1 tmt2 SUC4* sugar levels are more than twice that of WT. It is notable that *tmt1 tmt2 SUC4* showed only minor reduction in biomass compared to WT suggesting that a doubling of sugar content had little effect on photosynthesis. *suc2 adg1* and *suc2 adg1 tmt1 tmt2 SUC4* showed 6.9- and 7.3-fold lower starch content as compared to WT, respectively. There was no significant difference in starch content between WT and *tmt1 tmt2 SUC4*. These results highlight the efficiency of both SUC2-dependent phloem loading in transporting sugar out of leaves and ADG1-mediated starch biosynthesis in leaves. That rates of FA synthesis, total FA- and TAG accumulation are higher in *suc2 adg1 tmt1 tmt2 SUC4* than in *suc2 adg1* are apparently paradoxical because lower sugar levels are associated with increased FA synthesis rates, FA- and TAG accumulation. These sugar levels are averaged over the tissue and the FA and TAG accumulations likely reflect differences in the distribution of sugar between the cytoplasm and vacuoles in cells of the two lines. Specifically, in *suc2 adg1 tmt1 tmt2 SUC4*, vacuolar levels of sugar are expected to be lower than those of WT, and because the vacuolar volume is larger than that of the cytoplasm, sugar concentrations presented herein are an average of all sub-compartments rather than those of the cytoplasm. Indeed, given the much larger volume of the vacuole relative to the cytoplasm, a decrease of only 40% of total sugars in *suc2 adg1 tmt1 tmt2 SUC4* relative to *suc2 adg1* is likely the consequence of a substantial increase in cytoplasmic sugar levels in *suc2 adg1 tmt1 tmt2 SUC4* consistent with the higher FA synthesis rates, FA and TAG accumulation levels in this line.

The dramatic increase in FA synthesis and TAG accumulation in *suc2 adg1 tmt1 tmt2 SUC4* likely arises from two independent sugar-dependent mechanisms. The first is a direct metabolic effect in which sugars provides carbon skeletons that are converted to pyruvate and subsequently to acetyl-CoA, the substrate of the first committed step in FA synthesis, acetyl CoA carboxylase. Thus, increased levels of sugars are expected to result in increased FA synthesis. The second mechanism involves sugar signaling, i.e., the regulation of FA synthesis by sucrose non-fermenting 1–related protein kinase 1 (SnRK1) (Price et al., 2004; Li and Sheen, 2016). Under low sugar conditions, SnRK1 kinase phosphorylates WRINKED1 (WRI1), a master transcriptional activator of genes involved in glycolysis and FA synthesis (Maeo et al., 2009). Phosphorylated WRI1 is rapidly degraded by the ubiquitin/proteasome pathway, thereby downregulating FA synthesis. Under high sugar conditions such as those in the leaves of *suc2 adg1* and *suc2 adg1 tmt1 tmt2 SUC4*, SnRK1 is inhibited, preventing WRI1 phosphorylation (Zhai et al., 2021). Stabilized WRI1 strongly induces FA synthesis (Zhai et al., 2017a). A similar SnRK1-dependent mechanism regulates the stability of DGAT1, a factor often considered to be rate limiting for the incorporation of FA into TAG. Indeed, in both *suc2 adg1* and *suc2 adg1 tmt1 tmt2 SUC4*, only 31 and 46% of the FA that accumulated beyond the levels found in the membranes of WT were incorporated into TAG, suggesting a deficit in TAG assembly.

The accumulation of TAG in vegetative tissues is commonly associated with a decrease of size of the engineered plants in this and former studies and in tobacco (Vanhercke et al., 2017) and in sugarcane (Zale et al., 2016). The decrease in size associated with sucrose phloem loading in *suc2* is particularly severe, and we previously reported that it can be partially mitigated by increasing growth of both shoots and roots by combining with *adg1* (Zhai et al., 2017b), which blocks starch synthesis thereby increasing sugar levels and stimulating TAG accumulation by the mechanisms described above. The incremental increase in size in *suc2 adg1* was attributed to elevated sugar levels that drove both FA synthesis and increased passive outward sugar leakage into the phloem and its subsequent transport to growing roots and shoots fueling increased growth and development. The further incremental increase in plant size and root length seen for *suc2 adg1 tmt1 tmt2 SUC4* relative to *suc2 adg1* in this study likely reflects an additional increase in the levels of cytoplasmic sugars at the expense of vacuolar sugars resulting from the suppression of TMT1 and TMT2 and overexpressing SUC4. Recently, Beechey-Gradwell et al. demonstrated that enhanced accumulation of leaf lipid in ryegrass also increases photosynthesis rates and biomass (Beechey-Gradwell et al., 2020). Higher lipid accumulation and fatty acid synthesis rate in *suc2 adg1 tmt1 tmt2 SUT4* may also contribute to its size increase.

An interesting aspect of this study was that polyunsaturated fatty acids were significantly elevated in *suc2 adg1* and *suc2 adg1 tmt1 tmt2* overexpressing *SUC4*, suggesting upregulation of the oleate desaturase FAD2. We previously demonstrated that the elevated sugar levels in *suc2 adg1* result in the stabilization and accumulation of WRI1 by inhibiting SnRK1-dependent phosphorylation of WRI1 (Zhai et al., 2017a). A recent developmental study of *Prunus sibirica*, a novel woody biodiesel feedstock, demonstrated the coregulation of WRI1 and FAD2 (Deng et al., 2019). In Arabidopsis, WRI1 was reported to directly bind to a site upstream of the FAD2 open reading frame (Kim et al., 2016). Together these observations present a possible link between the elevated levels of sugar and increase in polyunsaturated fatty acids observed in this study.

## Conclusions

This study utilizes the model plant Arabidopsis to understand the consequences of altered sugar partitioning between the vacuole and the cytoplasm with respect to FA synthesis and TAG accumulation, and thus our hypothesis of remobilizing sugars from the vacuole to the cytoplasm to increased FA synthesis and TAG accumulation was validated. Blocking sugar movement into the phloem by impairment of *SUC2* is a useful way to explore the effects of increased levels of leaf sugar, but its consequence of starving growing parts of the plant for sugar enviably leads to dwarfing. The partial mitigation of this phenotype by inclusion of *adg1, tmt1, tmt2*, and the overexpression of *SUC4*, provide useful insights into the metabolic availability of sugar, and provides support for the use of similar strategies of relocalizing vacuolar sugar to the cytoplasm to drive increased FA synthesis and TAG accumulation in high stem sugar-accumulating crops such as sugarcane. The observation that much of the synthesized FA in *suc2 adg1 tmt1 tmt2 SUC4* is not converted to TAG suggests that overexpression of additional acyltransferases such as DGAT or PDAT, or others could further increase TAG accumulation. In addition, suppression of TAG degradation by downregulating the major TAG lipase, SDP1, or overexpressing TAG protection factors such as OLEOSIN1 might be useful in further optimizing TAG accumulation.

## Materials and Methods

### Plant Materials and Growth Conditions

Columbia-0 (the parental Wild-type), *tmt1 tmt2* (Schulz et al., 2011), *SUC4* overexpression line (Schulz et al., 2011), *suc2 adg1* (Zhai et al., 2017b) in *Arabidopsis thaliana* were used in this study. The homozygous *suc2 adg1 tmt1 tmt2* overexpressing *SUC4* was generated by crossing the corresponding parents. The homozygosity was verified by PCR genotyping using the primers shown in Table 1. All genotypes were grown in a light/dark cycle of 16h/ 8h with a day/night temperature of 23/19°C with 75% humidity and at a photon flux density of 250 mmol m^−2^ s^−1^.

**Table 1:** Primers used.

**Gene**	**Primer Pair Sequences (5^**′**^-3^**′**^)**	**Purpose**
*TMT1*	GGCAAAGCTTTCTCATTTCCTCGTAATTG	Genotyping for *tmt1*
*TMT1*	AACGAGGAGACTCGGGCAAATAAAACACC	Genotyping for *tmt1*
LB-3	TAGCATCTGAATTTCATAACCAATCTCGATACA	Genotyping
*TMT2*	GGAAATGCAGTTCTCAGGCATCAACG	Genotyping for *tmt2*
*TMT2*	GAGAAGAAGCGAGGAAAGACGCTGAATTG	Genotyping for *tmt2*
*SUC4*	GGTCAGTATGGGTGCACTTGG	Genotyping for *SUC4*
*SUC4*	GGTGAATTCCCACCTCCAAACAG	Genotyping for *SUC4*
*SUC2*	GTTTTTCGGAGAAATCTTCGG	Genotyping for *suc2*
*SUC2*	CAAATGCTGGAATGTTTCCAC	Genotyping for *suc2*

### Leaf Iodine Staining and Starch Quantification

For leaf starch iodine staining, fresh leaves were sampled from WT, *suc2-4* and *adg1-1suc2-4* and decolored by incubation in absolute ethanol at 70°C for 5 min before staining with diluted Lugol's solution (Tsai et al., 2009). For quantification, starch was extracted from equivalent amount of fresh leaf tissue sample across the genotypes in 1 mL of DMSO and 250 μL of 8 M HCl (Takeshita et al., 2015). Samples were incubated at 60°C for 30 min. Sample were diluted to 5 mL with deionized water and the pH was adjusted to between 4 and 5 before measuring starch using Lugol's solution (Kawano, 2015). Pure corn starch was used as reference standard for quantification.

### Leaf Sucrose, Fructose, and Glucose Quantification

Sucrose (SCA 20), Fructose (FA 20) and Glucose Assay Kits (GAGO 20) were obtained from Sigma Chemical Company, St Louis MO. Leaf sucrose, fructose and glucose assays were performed according to the manufacturers' protocols using supplied pure sugars as reference standards for quantification.

### Lipid Quantification

Total lipids were extracted from 100 mg of fresh leaf tissue into 700 μL of extraction buffer (methanol:chloroform:formic acid 2:1:0.1; v/v/v). Samples were vortexed vigorously for 60 min. Total lipids were extracted as the lower phase by adding 350 μL of 1 M KCL and 0.2 M H_3_PO_4_ followed by briefly vortexing and centrifuging at 2,000 × g for 10 min. For total FA quantification, 10 μL of the lipid fractions were transmethylated at 80°C for 90 min using 1 mL of boron trichloride-methanol. For TAG quantification, 60 μL of lipid fractions were separated by thin-layer chromatography on silica Gel 60 plates (Merck) using hexane:diethyl ether:acetic acid (70:30:1; v/v/v) as the development solvent. Lipids were visualized after spraying developed plates with 0.05% pirmuline in 80% acetone. TAG fractions were identified by illuminating the stained TLC plate under UV light. The TAG bands were scraped from the TLC plate and transmethylated as described above for total fatty acids. Five microgram of C17:0 internal standard was added prior transmethylation to permit absolute quantitation for both the assays. 2 mL of 1:1 Hexane:water was used to extract FA methyl esters and the resulting lipids were dried under liquid nitrogen. Prior to analysis by gas chromatography, samples were re-dissolving in 100 μL of hexane. Fatty acid profile was quantified using an Agilent Technologies 7890A gas chromatography system coupled to a 597C mass detector and equipped with a DB23 column (Supelco; 60-m X 0.25-mm). Lipid samples were separated using capillary column (Agilent J & W DB-23, 30 m × 0.25 μm × 0.25 μm). The oven temperature was programmed to increase temperature from 100 to 240°C at a rate of 12.5°C/min. The identities of individual FA were confirmed by mass spectrometry of their methyl esters.

### *In vivo* [1-^14^C] Acetate Labeling

[1-^14^C] acetate labeling was performed according to the method of Koo et al. (2004). In brief, half leaves were incubated in 25 mM Na-Morpholinoethanesulfonic acid (MES, pH 5.7) buffer containing 0.01% w/v Tween-20 as wetting agent under illumination (180 μmol photos m^−2^s^−1^) at 25°C. Labeling was initiated by the addition of 10 μCi of sodium [1-^14^C] acetate solution (58 mCi/mmol, American Radiolabeled Chemicals). Labeling was terminated by the removal of the medium after which the leaf material was washed 3x with deionized distilled water. Total lipids were extracted and separated as described above. Lipids were suspended in 2 mL of Ultima Gold liquid scintillation cocktail (PerkinElmer) and radioactivity was quantified in CPM using scintillation counter (Packard BioScience).

## Data Availability Statement

The raw data supporting the conclusions of this article will be made available by the authors, without undue reservation.

## Author Contributions

Work conceived and designed by SA and JS. Experiments and data analysis were carried out and interpreted by SA, HL, JK, ZZ, and JS. SA and JS wrote the paper with contributions from all co-authors. All authors contributed to the article and approved the submitted version.

## Author Disclaimer

The views and opinions of authors expressed herein do not necessarily state or reflect those of the United States Government or any agency thereof.

## Conflict of Interest

The authors declare that the research was conducted in the absence of any commercial or financial relationships that could be construed as a potential conflict of interest.

## Publisher's Note

All claims expressed in this article are solely those of the authors and do not necessarily represent those of their affiliated organizations, or those of the publisher, the editors and the reviewers. Any product that may be evaluated in this article, or claim that may be made by its manufacturer, is not guaranteed or endorsed by the publisher.
